# Occurrence and Multidrug Resistance in Strains of *Listeria monocytogenes* Recovered from the Anaerobic Co-Digestion Sludge Contained in a Single Stage Steel Biodigester: Implications for Antimicrobial Stewardship

**DOI:** 10.3390/microorganisms11030725

**Published:** 2023-03-11

**Authors:** Christy Echakachi Manyi-Loh, Anthony Ifeanyin Okoh, Ryk Lues

**Affiliations:** 1 Centre of Applied Food Sustainability and Biotechnology (CAFSaB), Central University of Technology, Bloemfontein 9301, South Africa; 2 SAMRC Microbial Water Quality Monitoring Centre, University of Fort Hare, Alice 5700, South Africa; christymanyiloh5@gmail.com; 3 Department of Environmental Health Sciences, College of Health Sciences, University of Sharjah, Sharjah P.O. Box 26666, United Arab Emirates

**Keywords:** anaerobic co-digestion, swine manure/pine wood sawdust, *Listeria* species, antibiotic resistance, multiple antibiotic resistance (MAR) index

## Abstract

*L. monocytogenes* is a zoonotic foodborne pathogen with inherent adaptability to tolerate environmental and physiological stresses, thereby causing severe disease outbreaks. Antibiotic resistant foodborne pathogens are a challenge to the food industry. A total of 18 samples were pooled from a bio-digester co-digesting swine manure/pinewood sawdust, and evaluated for the occurrence of bacterium plus total viable counts using the spread plate method. The recovered bacterial isolates were presumptively identified by growth on selective medium and confirmed by biochemical characterisation, leading to the isolation of 43 *L*. *monocytogenes*. The isolates were characterized based on their susceptibility to antibiotics via the Kirby-Bauer disc diffusion technique against a panel of 14 antibiotics. Equally, the multiple antibiotic resistance (MAR) index was calculated, and MAR phenotypes generated. The bacterial counts were between 10^2^ and10^4^ cfu/mL. Complete susceptibility (100%) was demonstrated to ampicillin, gentamicin and sulfamethoxazole, which are the drugs of choice in the treatment of listeriosis. In addition, intermediate sensitivity occurred at 25.58% to cefotaxime, and the highest resistance (51.16%) was exhibited against nalidixic acid. The MAR index ranged from 0 to 0.71. Overall, 41.86% of the Listeria isolates displayed multidrug resistance, with 18 different MAR phenotypes, demonstrating CIP, E, C, TET, AUG, S, CTX, NA, AML, NI as the greatest MAR phenotype. It can be concluded that the isolates yielding MAR > 0.2 originated from the farm, where antibiotics had been in routine use. Therefore, strict monitoring of antibiotics use in the farm is crucial to mitigate further increase in antibiotic resistance amongst these bacterial isolates.

## 1. Introduction

*Listeria* species are described as Gram-positive, facultatively anaerobic, psychrotrophic, rod-shaped, non-spore-forming bacteria. Of the twenty (20) species categorised under the genus *Listeria*, only *L. monocytogenes* and *L*. *ivanovii* exist as pathogens, and the former is the bacterial pathogen responsible for listeriosis in humans and other mammals [1]. *Listeria* species are ubiquitous in nature (found in water, soil, vegetation, etc.) [2] owing to their ability to thrive in harsh environmental conditions, including low and high pH environments, low temperature, and high salt concentration, ultraviolet light and the presence of heavy metals and biocides [2]. The bacterium tends to acquire tolerance to several physical and physicochemical stresses [3]. In precise terms, several authors have demonstrated the occurrence of *L*. *monocytogenes* in animal manure and wastewaters [4]. Pourcher et al. [5] noted a great prevalence of *L. monocytogenes* in pig manure kept in pits and in lagoons, following biological treatment. Desneux and colleagues [6] demonstrated the alteration of the cultivability of *L*. *monocytogenes* during storage of manure, transforming into a viable but non culturable state. Thus, humans might become contaminated with the bacterium upon consumption of dairy products, meat products and via handling or practices that occur within farms, hence leading to listeriosis infection [7].

Proper diagnosis of listeriosis precedes appropriate treatment, and the gold standard of isolating *Listeria monocytogenes* from environmental samples involves selective enrichment of the microbiological medium to facilitate successful culture. In addition, treatment of *Listeria* infections requires the employment of antibiotics that elicit rapid and bactericidal action against *L*. *monocytogenes,* thereby eradicating the pathogen and healing the individual [8]. This favourable outcome is reliant upon the immediate administration of antibiotics [9]. In this light, Pagliano et al. [8] mentioned that the treatment of listeriosis in humans involves gentamicin, amoxicillin/ampicillin, penicillin, chloramphenicol, tetracycline, rifamycin or trimethoprim and sulfamethoxazole as a standalone (single) or a combination therapy. Therefore, the choice of the correct antibiotic with the appropriate bactericidal action requires monitoring the distribution of antibiotic resistance among the *Listeria* species that occur in a particular region [10]. 

From a global perspective, antimicrobial resistance had been viewed as a tangible and developing menace to the health of the population worldwide. The origin of bacterial resistance to the use of antimicrobials has been ascribed to the overuse and imprudent use of antimicrobials, such as medications for humans and in veterinary medicine [11]. Of great concern, and of more critical consideration, is the development of bacterial pathogens with resistance to antimicrobials, since they have been known to compromise the efficacy of treatment regimens for human infections that manifest with less severe conditions, and even severe conditions that are life threatening [11]. Clearly, the acquisition of a vast number of antibiotic resistance genes seems to be occurring rapidly in bacterial strains, and many of the said genes may arise from organisms inhabiting food, or the gastrointestinal tracts of animals, and are living as commensals [12]. Accordingly, earlier studies conducted by Morena and co-authors [13] as well as Sanlibaba and colleagues [14] demonstrated that *Listeria* species may develop resistance to different antibiotics, including oxacillin, clindamycin, fosfomycin, ampicillin, tetracycline and nalidixic acid. *Listeria* species acquire antibiotic resistance which varies widely amongst the strains, according to the source and the year of isolation, antimicrobial use in both humans and animals, and geographical differences [10]. Therefore, monitoring of changes in antibiotic resistance, which occurs in *L*. *monocytogenes* and other *Listeria* species, becomes obvious as the emergence and dissemination of resistant strains pose serious threats to human health.

It is worth mentioning that several studies of antimicrobial resistance, relating to the bacterial organism, *L*. *monocytogenes* have been based on human isolates; however, broadening the method with observation data obtained from diverse samples, comprising of foods, food-producing animals, food producing environment and animal manure, is crucial [15]. Due to its easy spread in the environment, its virulence, and its transmission via employees, raw material and equipment in the food processing environment, the bacterium is provided with factors ensuring its ability to adapt to environmental conditions, causing long-lasting colonisation. Consequently, *L*. *monocytogenes* can persist in different habitats and can be recovered from food samples, the farm environment and from the food processing/production environment [16]. Wiśnierwski et al. [17] noted the serotype 1/2a was prevalent in *L*. *monocytogenes* isolates recovered from food, while the serotype 1/2c occurred among strains obtained from food processing environment, and the food strains demonstrated greater resistance (92.3%) to clindamycin compared to 82.5% resistance displayed by the strains from the food processing environment. Moreover, Muchaamba and co-authors [18] mentioned that the organism is genetically diverse, involving 14 serotypes, four major evolutionary genetic lineages, and several multilocus sequence types (MLSTs); therefore, in their study of food samples and samples from the food processing environment, the authors uncovered, via genome comparison of the *L*. *monocytogenes* strains, numerous, moderate differences in virulence and genes associated with stress between the strains. Muchaamba and colleagues [18] opined that these differences, as well as variations in gene expression, might greatly affect the virulence observed as well as phenotypic differences in stress sensitivity. 

Reflecting on the high death rate associated with listeriosis in susceptible populations (pregnant women, newborn, elderly, children and immunocompromised), it is essential to determine the efficacy of current antimicrobials and to preserve these, as well as closely observe the development of resistance in the *Listeria* strains to antimicrobials [10,19]. Studying resistance to antimicrobials in animal-borne and commensal bacteria occurring in food-producing animals and the food derived from these, is a first step to understanding the emergence and the dissemination of resistance, providing relevant risk assessment data, and evaluating targeted interventions [20].

To compound the problem of antibiotic resistance, recent studies have revealed the presence of multiple antibiotic resistance. Different mechanisms, and their combinations, are used by bacteria to develop resistance to antibiotics, and the occurrence of plasmids containing single or several resistance genes that encode single antibiotic resistance phenotype usually causes multiple resistance in bacteria. Bacterial pathogens associated with livestock and demonstrating resistance, including multidrug resistance, remain a major concern globally as they can be transmitted from animals to humans, causing foodborne and zoonotic diseases. Research findings demonstrated the possibility of contaminating foods with antibiotic resistant bacteria and genes during the continuum from farm to processing to retail to consumers [21]. According to a One Health approach, antibiotic and multidrug resistance originating from food animals can affect the health of humans and plants [22]. The authors demonstrated the presence of multidrug resistance amongst the 91.19% of zoonotic enteropathogens (*E*. *coli*, *Salmonella* sp., *Yersinia* sp., *Campylobacter* sp.) recovered from a codigesting sludge, comprising 75% pig manure and 25% pine wood saw dust, undergoing anaerobic codigestion at a psychrophilic temperature range. Baloyi and colleagues [23] noted the presence of multidrug resistance among 87.51% of *E*. *coli* strains recovered from apples, spinach, carrots, tomatoes and cabbage sold in open markets in Gauteng Province, South Africa. Mthembu et al. [24] observed multidrug resistance in forty-three percent (43%) of *Salmonella* species isolated from faecal and environmental samples obtained from livestock production systems in the country. Abdalla and colleagues [25] investigated the prevalence of diarrheaic *E*. *coli* isolates along the pig production continuum and revealed that seventy-three percent (73%) of these isolates were multidrug resistant. The authors concluded that food animals are potential reservoirs that can be implicated in the transfer of bacteria to humans; therefore, adherence to good hygienic practices along the pig production continuum is paramount in mitigating the risks associated with transmission and infection, and ensuring food safety. Sineke [26] in a study involving farm-to-fork in an intensive pig production chain in the uMgungundlovu district, Kwa-Zulu Natal, characterised eighty-four (84%) of *Staphylococcus aureus* as multidrug resistant. Therefore, antimicrobial resistance is rising, and concerns pertaining to antimicrobial resistance are increased because of the lack of the discovery of new antibiotics. Antibiotic resistance genes can be transferred to other bacteria of the same or different species [27]. Sanlibaba and colleagues [14] observed a 73.91% multidrug resistance in *L*. *monocytogenes* isolated from 190 raw meat samples collected in Ankara, Turkey. 

Keet and Rip [28] noted that the majority of the *L*. *monocytogenes* strains expressed multiple resistance (chloramphenicol, erythromycin, and tetracycline), in a study conducted in the Western Cape of South Africa, contradicting certain global resistance patterns. More strikingly, South Africa in 2018, witnessed the largest global listeriosis outbreak to date, wherein 674 persons were hospitalised, and 183 death cases reported. Surprisingly, there is a paucity of data on the bacterium’s resistance to antibiotics, from different samples across the country. It is relevant to determine the antibiotic resistance profiles of *Listeria* species obtained from animal (pig) manure at a piggery farm located in the Eastern Cape Province of the country to elucidate whether the patterns mirror the resistance patterns in the different regions of the country and elsewhere in the world.

The multiple antibiotic resistance (MAR) index of each bacterial isolate is usually calculated to estimate the measure of contamination, since the MAR index is described as an accurate, effective and cost-effective method employed in the source tracking of antibiotic resistant organisms. An MAR index value above 0.2 indicated a high risk of contamination in a region in which antibiotics are employed on a regular basis [29]. Our investigation sought to examine the presence of *Listeria monocytogenes* in samples from a co-digesting medium constituting swine manure and pine wood sawdust. The characterisation of the organism included the determination of antibiotic resistance patterns and multidrug resistance profiles, as well as indices of forty-three *Listeria* species that were recovered from the samples. Implications in antimicrobial stewardship are outlined.

## 2. Materials and Methods

### 2.1. Sampling

An anaerobic single-stage steel biodigester of 100 L capacity with a stirrer was designed. The samples were fresh, undiluted pig manure and pine wood saw dust procured from a piggery farm and a sawmill located very close to the University of Fort Hare, Alice Campus. The samples were pre-treated and mixed in the ratio of 3:1 to produce a slurry, in water in an equal volume. The slurry was fed into the biodigester, which was batch operated at a psychrophilic and mesophilic temperature range (13.16–29 °C) over a period of seven (7) months [30]. Physicochemical parameters (pH and temperature) were monitored throughout the process. The pH of every withdrawn sample was measured using a PHSCAN 30 pH meter, while different temperature sensors were introduced at different levels/locations of the slurry to record temperature and were externally connected to a Hobo U12data logger, configured to log every five (5) minutes [31].

Prior to sample collection, the digesting mixture in the digester was stirred gently (approximately 2–3 min) in a uniform manner to ensure even distribution of the microorganisms and temperature throughout the digesting substrates using the stirrer [32]. Subsequently, between 5 and 7 mL of the digesting substrate was collected from several locations of the biodigester and pooled to represent the daily sample. Each sample was introduced into tryptic soy broth (Liofichelm, Diagnostics, Roseto degli Abruzzi, Italy) contained in a sterile centrifuge tube, which was clearly labelled and transported on a cold chain to the laboratory for immediate processing [33].

Overall, eighteen pooled samples were collected, and consisted of both untreated biomasses (portion of the prepared slurry collected prior to charging of the digester and denoted as the day 0 sample) and treated biomasses (samples that were withdrawn from digester following charging after days of commencement of the anaerobic digestion process, i.e., from samples 2 to 18). The samples were collected every 7 or 14 days.

### 2.2. Bacterial Culture for Isolation

For the primary isolation of *L*. *monocytogenes*, each sample was cultivated on *Listeria* Selective Oxford Agar (Conda, Madrid, Spain) that was incorporated with Listeria selective supplement (LSOA; Oxoid, Basingstoke, UK) reconstituted in acetone/water in the ratio of 1:1. In detail, each sample (1 g) was ten-fold serially diluted in 9 mL of 0.9% sterile physiological saline contained in labelled sterile test tubes to constitute dilutions from 10^−1^ to 10^−5^. The dilution process was performed to reduce bacterial concentration in order to achieve growth of distinct colonies during incubation, thereby permitting easy enumeration and ensuring purity. The medium (LSOA) was prepared according to manufacturer’s instructions. One-hundred microlitres of each dilution from 10^−1^ to 10^−5^ was inoculated onto solidified LSOA plates through the spread plate technique as per the method of Rowbotham and Ruegg [34].

Following inoculation, the plates were incubated at 37 °C for 24–48 h. Subsequently, the growth of bacterial colonies was examined, and the number of emergent colonies on respective tables was counted and recorded. *L*. *monocytogenes* was identified as grey colonies surrounded by black halos (esculin hydrolysis) and counted as colony forming units per millilitre [35]. Each measurement represented the mean of triplicate experiments.

### 2.3. Purification and Preservation of Bacterial Strains/Colonies

To achieve purity of the bacterial isolates, single, distinct or well-isolated colonies were each sub-cultured on individual Mueller Hinton agar (Conda, Madrid, Spain) plates supplemented with measured volumes of the *Listeria* selective supplements (Oxoid, UK) and reconstituted in acetone/water solvent in the ratio 1:1. Each isolate was sub-cultured several times on Mueller Hinton agar plates to ensure purity as previously reported by Manyi-Loh et al. [36]. The sub-cultured plates were incubated for 24–48 h at 37 °C. After incubation, an appreciable quantity of the bacteria was transferred into sterile tryptic soy broth (Liofichelm, Diagnostics, Roseto degli Abruzzi, Italy) enriched with 20% glycerol in cryovials and stored at −80 °C for preservation until further analysis.

### 2.4. Identification of L. monocytogenes Isolates

A presumptive identification of the bacterium was based on its cultivation on selective medium, i.e., *Listeria* Selective Oxford Agar (LSOA; Conda, Spain), modified with calculated volumes of *Listeria* selective supplements (Oxoid, UK) reconstituted in acetone/water solvent in the ratio 1:1. *L*. *monocytogenes* isolates were observed after growth on the medium as tiny to small grey colonies surrounded by black halos. Therefore, presumptive identification was based on morphological and cultural characteristics on the selective agar. These isolates were confirmed through the performance of biochemical tests, including the expression of enzyme activities (catalase, urease, oxidase), the indole reaction, and the ability to ferment sugars without the production of hydrogen sulphide gas. These tests were performed following the procedures of Cheesbrough [37]. Of the 74 presumed *L*. *monocytogenes* strains, 43 strains were biochemically confirmed as *L*. *monocytogenes,* as they presented with negative urease and oxidase abilities, negative indole reaction, and positive reaction for catalase, as well as the ability to degrade lactose and D-glucose without any gas production. These isolates were employed in subsequent antibiotic susceptibility testing.

### 2.5. Susceptibility Testing with Conventional Antibiotics

In determining the resistance of the *Listeria* species against a suite of 14 antibiotics, the Kirby Bauer disc diffusion method was adopted according to the procedures previously described by Sanlibaba et al. [38]. The choice of the antibiotics was based on their use in both human and veterinary medicine and included the following: ampicillin (25 μg/disc), augmentin (30 μg/disc), erythromycin (15 μg/disc), tetracycline (25 μg/disc), ciprofloxacin (5 μg/disc), nalidixic acid (30 μg/disc), streptomycin (300 μg/disc), gentamicin (10 μg/disc),cotrimoxazole (25 μg/disc), chloramphenicol (30 μg/disc), cefotaxime (30 µg/disc), sulfamethoxazole (100 μg/disc) nitrofurantoin (300 µg/disc) and amoxicillin (10 µg/disc) (Mast Diagnostics Limited, Bootle, UK). The procedure employed Mueller Hinton agar (Conda, Spain) that was incorporated with appropriate volumes of *Listeria* selective supplement (Oxoid, UK) reconstituted in acetone/water in a ratio of 1:1. The medium was prepared following the manufacturer’s instructions and dispensed aseptically into sterile petri dishes (Merck, Lethabong, South Africa) to solidify. Bacterial strains were sub-cultured on Mueller Hinton agar plates (Merck, South Africa), from which accurate quantities of growth were transferred into 0.9% physiological saline contained in well labelled sterile test tubes to create individual inocula with a bacterial concentration that corresponded to a 0.5 Mac Farland standard (approximately 10^8^ cfu/mL). The antibiotics were introduced onto inoculated Mueller Hinton agar plates (MHA) according to the protocols of the Clinical Laboratory Standard Institute [39]. After incubation for 24–28 h, the diameter of inhibition zones was measured to the nearest mm, and interpreted and categorised as susceptible, intermediate or resistant to a particular antibiotic based on the recommendations outlined by CLSI [39]. The breakpoints for sulfamethoxazole/trimethoprim and ampicillin were adopted from CLSI [39], while the breakpoints for *Staphylococcus* were considered to complement the remaining antibiotics according to Du et al. [40]. Each measurement represented triplicate assays. *Escherichia coli* ATCC (American Type Culture Collection) 25922 was employed as the quality control strain throughout all the assays.

### 2.6. Calculation of Multiple Antibiotic Resistance (MAR) Index of Bacterial Strains

The determination of the MAR index was conducted using a method adopted from Joseph et al. [41], i.e., the number of antibiotics to which each bacterial strain is resistant to represented as (a) divided by the overall number of antibiotics considered in the investigation denoted as (b). The formula is MAR = a/b. A MAR index value above 0.2 indicates the isolate originates from a region where it has been routinely exposed to antibiotics, and can be referred to as multidrug resistant, and a MAR index value lower than 0.2 indicates that the isolate originates from a source/region where antibiotics are seldomly used.

### 2.7. Statistical Analysis

Microsoft Excel was used to calculate the means of zone diameters and percentages of susceptible and resistant strains as well as to construct figures on the prevalence of antibiotic resistance/susceptible rates. Descriptive statistics and frequencies were employed.

## 3. Results

### 3.1. Monitoring the Parameters (pH and Tempertaure) of the Anaerobic Digestion Process

**Figure 1:**
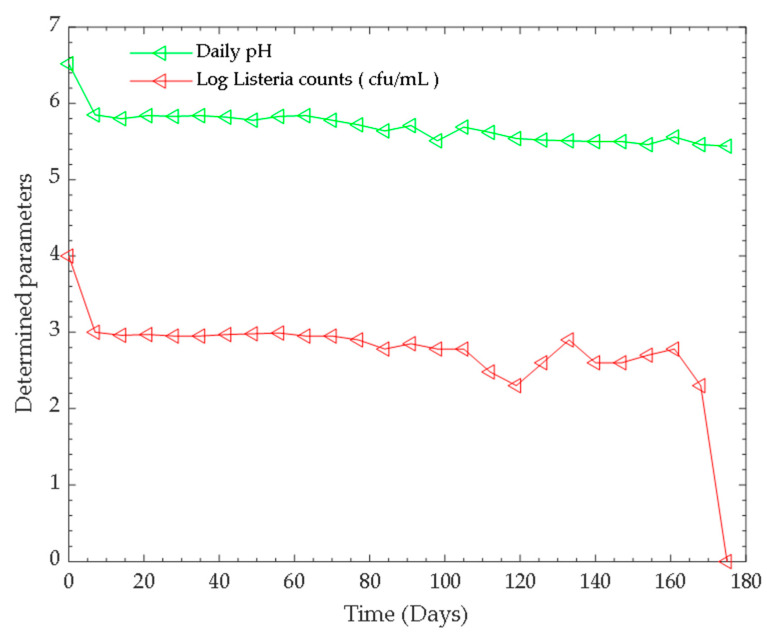
Log bacterial counts and daily pH over time.

**Figure 2:**
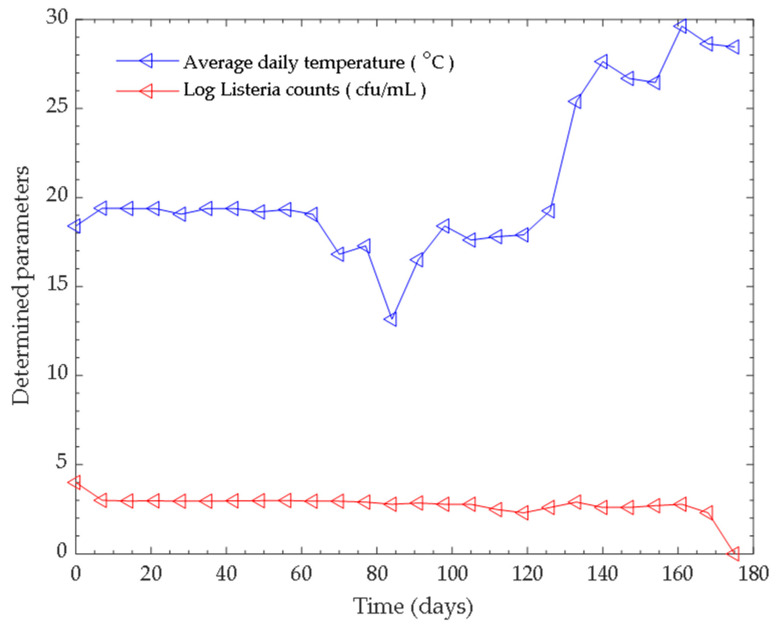
Log bacterial counts and average daily temperature over time.

Anaerobic co-digestion occurred over a pH range of 5.46–6.52, and the temperature of the process was regulated at both psychrophilic and the mesophilic temperature ranges through the seven months period. The temperature range within the first five (5) months was termed as psychrophilic phase temperature, while in the latter two months, the temperature range of the process was described as the mesophilic phase temperature. The influence of pH and temperature on the log viable counts of *Listeria monocytogenes* is shown in Figure 1 and Figure 2.

### 3.2. Bacterial Counts and Identification 

Viable *Listeria* counts ranged from 2.3 × 10^2^ to 1 × 10^4^ cfu/mL. A total of 18 pooled samples were withdrawn from the bio-digesting chamber every 7 or 14 days. All the samples (18/18) were found positive for *L*. *monocytogenes*, yielding a 100% prevalence rate. The bacterium was enumerated alongside four other bacteria, belonging to the Gram-negative category, though the data are not reported in this study. However, the viable counts of *L*. *monocytogenes* bacterium gradually decreased over time as the anaerobic digestion process progressed by 1Log reduction (90% reduction), and the bacterium experienced the longest survival time of 175 days in the biodigester amongst the Gram-negative bacteria investigated (*E*. *coli*, *Salmonella* sp, *Yersinia* sp. and *Campylobacter* sp.). From these viable bacterial counts, a total of 74 bacterial isolates were presumptively identified as *Listeria* species. Only 43 isolates were biochemically confirmed as demonstrating esculin hydrolysis, with catalase enzyme activity, fermenting glucose and lactose without the production of hydrogen gas, lacking the presence of urease and oxidase, and were negative in the indole test, yielding a recovery rate of 58.12%.

### 3.3. Antibiotic Susceptibility Testing

Overall, the *Listeria* species demonstrated varied sensitivity to 14 antibiotics belonging to ten different antibiotic classes. Table 1 shows the frequency of the *Listeria* isolates that were susceptible to the 14 investigated antibiotics. The sensitivity to antibiotics depended on the isolates and the chemical nature of the antibiotics; nine isolates (20.93%) were susceptible to the complete antibiotics panel employed in the investigation. In precise terms, the greatest susceptibility of the isolates in terms of frequency was displayed toward ampicillin, gentamicin and sulfamethoxazole (100%), co-trimoxazole (97.6%), augmentin (93.02%), and streptomycin (90.7%). The different proportions of the *Listeria* species demonstrating intermediary sensitivity were in the order: 25.58% (cefotaxime), 23.26% (ciprofloxacin) and 13.95% (tetracycline). 

**Figure 3:**
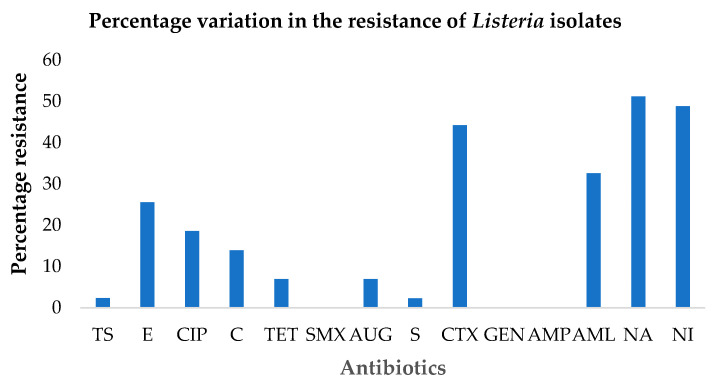
Frequency of *Listeria* isolates exhibiting resistance against the tested antibiotics (TS, co-trimoxazole; E, erythromycin; CIP, ciprofloxacin; C, chloramphenicol; TET, tetracycline, SMX, sulfamethoxazole, AUG, augmentin; S, streptomycin; CTX, cefotaxime, GEN, gentamicin; AMP, ampicillin; AML, amoxicillin; NA, nalidixic acid; NI, nitrofurantoin).

The lowest antibacterial activity (the highest resistance displayed by the isolates) was exerted by nalidixic acid (51.16%), nitrofurantoin (48.84%) and tetracycline (44.19%) as shown in Figure 3. In addition, 11 isolates (25.58%) showed resistance to only one antibiotic that was either cefotaxime (eight isolates) or amoxicillin (two isolates) or erythromycin (one isolate). 

**Figure 4:**
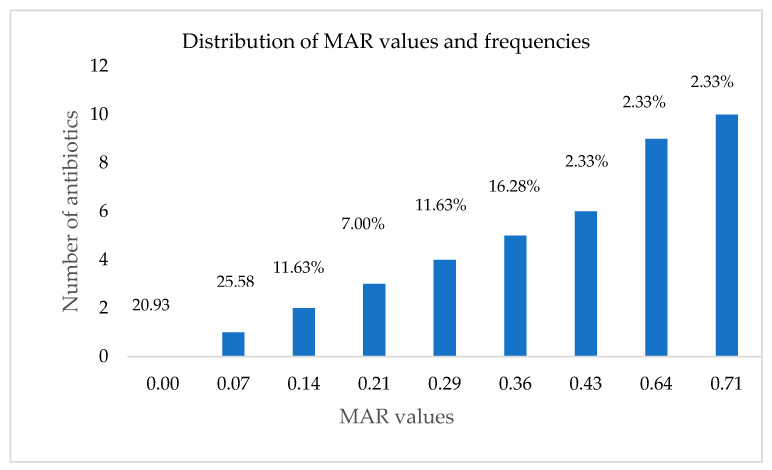
Distribution of MAR values and frequencies of Listeria isolates in the study.

The data in Figure 3 (only for resistance) was employed to determine the index of multiple antibiotic resistance (MAR) for each bacterial strain. MAR values ranged from 0 to 0.71. MAR value > 0.2 denote multidrug resistance and includes bacterial isolates demonstrating resistance to three or more antibiotic classes. In this study, the number of *Listeria* species with MAR values > 0.2, was 18 of 43, i.e., a prevalence rate of 41.86%, as seen in Figure 4. 

Multiple resistance to antibiotics (MDR) in the isolates was further investigated based on the categories of the antibiotics evaluated in the investigation. The different MAR phenotypes identified in this study are presented in Table 2. A total of 18 MAR phenotypes was identified. It was observed that only one isolate exhibited resistance to ten tested antibiotics (MAR = 0.71), which was expressed phenotypically as NA, NI, AML, CTX, CIP, TET, AUG, E, C, S, whereas the MDR (multidrug resistance) trait was prevalent in nine different antibiotic classes, including penicillins, quinolones, macrolides, amphenicols, tetracyclines, aminoglycosides, polypeptides, cephalosporin and nitrofurans. Another, nine antibiotics (MAR = 0.64), as well as one other isolate resistant to six antibiotics (MAR = 0.43) were represented by the MAR phenotypes NA, NI, AML, CTX, CIP, AUG, COT, E, C and NA, NI, CTX, AUG, E, C, respectively. 

As mentioned previously, one isolate had the highest MAR index value of 0.71, followed by another isolate with an MAR index of 0.64, showing elevated level of resistance to antibiotics in these isolates; thereby clearly indicating that the pig farm from which the swine manure was procured harboured *Listeria* species in its environment that were multidrug resistant, creating a great likelihood of entering the food chain through the animals. Furthermore, the highest frequency of MAR was observed against five antibiotics denoted by a MAR value of 0.36 with an occurrence of 16.23% (i.e., demonstrated by seven isolates), and were represented by varied MAR phenotypes, followed by resistance to four antibiotics and then three antibiotics with frequencies and MAR values of 11.63% and 0.29, and 7.0% and 0.21, respectively (Table 2).

## 4. Discussion

*Listeria monocytogenes* is ubiquitous in nature, with occurrence in vegetation, soil, agricultural environment (animal feed, meat, and dairy products), water, sewage and the excreta of human and animals, and is a food-borne pathogen [2]. We investigated its occurrence in swine manure blended with a small fraction of pine wood sawdust that was prepared for anaerobic co-digestion in a single stage steel biodigester to produce biogas for the sanitisation of the waste prior to disposal into the environment. By monitoring the process for efficient performance of the system, we enumerated *Listeria monocytogenes* counts over time and isolated and identified the organism, as well as conducting profiling of the antibiotic resistance and multidrug resistance behaviour/responses. With respect to data available in the public and science domains, following the outbreak of a *Listeria* infection that occurred in South Africa in 2018, our study is amongst early studies that have investigated the occurrence and multidrug resistance in strains of *Listeria* species recovered from co-digested mixture of animal (swine) manure collected from a pig farm.

Viable counts ranged from 2.3 × 10^2^ to 1 × 10^4^ cfu/mL, indicating the potential of the manure to cause pollution. Details of the findings, describing the effect of time on the dynamics of the bacterium have been published, together with those of other organisms (Gram-negative bacteria) by Manyi-Loh and Lues [22]. The authors observed a 1 log reduction of *L*. *monocytogenes*, compared to the other bacteria investigated. A period of 175 days, which was noted as the longest survival period, as opposed to 77 days for *E*. *coli*, 84 days for *Salmonella* sp, 98 days for *Yersinia* sp. and 112 days for *Campylobacter* spp. This is explained by the fact that the cell wall of *L*. *monocytogenes* is constituted of several layers of peptidoglycan and teichoic acids, contributing to its protection and resistance against stress and environmental conditions [42], conferring the ability to tolerate life-threatening environmental conditions, including a wide range of pH values, broad temperature ranges, and deficiencies in water, in addition to the presence of other metabolites/compounds and conditions that occur following the anaerobic digestion process [43,44]. Wiśniewski et al. [17] remarked that subjection of *Listeria* species to environmental stress conditions during food production has a great impact on its pathogenicity, gene expression and changes in antimicrobial resistance.

The growth and survival of micro-organisms can be influenced by environmental factors including water, temperature, pH, and nutrients. pH and temperature are interdependent, and both exert a great influence on the generation time and lag phase of bacteria. In biogas technology, pH and temperature are amongst the factors affecting the anaerobic digestion process [45]. The optimum pH necessary for optimum biogas yields lies between 6.5 and 7.2. Generally, bacteria can be categorised based on their optimal pH range for growth and multiplication into acidophiles (grow best below pH 5), neutrophiles (optimum growth at pH range 6–8 or between 5 and 9) and alkaliphiles (best growth above pH 9) [46]. Different bacterial species prefer different pH values. pH is an index of hydrogen ion concentration, related to the chemical activity of protons, and affects environmental conditions necessary for the growth and survival of microorganisms [47]. Saraswat and co-authors [47] noted that the development of bacteria during the anaerobic digestion process is affected by pH, which depends on carbon dioxide and volatile fatty acids. This is because pH interferes with the metabolisms of microorganisms [46]. In general, *L*. *monocytogenes* grows optimally at a pH ranging from 6 to 7 [48]; however, owing to its ubiquitous nature, the organism encounters different pH environments in the soil, acidic food, in the gastrointestinal tract of humans, and in animal manure. Most of these micro niches are described as acidic. The viability and survival of an enteric microbe is more challenging when living outside the host organisms [49]. In an environment, where the concentration of protons is high (low pH), the environment is acidic and the bacterial cells respond appropriately, making sure that the macromolecules and metabolic processes are adequately protected to sustain life. They respond by preventing a drop in intracellular pH below a threshold level necessary for viability [50]. Microbes can alter the pH around them since they perform biochemical reactions that involve the turnover of protons. In a bioengineered environment such as a biodigester, the pH over which the anaerobic digestion occurred in this study, was in the range 5.45–6.52 (Figure 1), and varies due to the series of biochemical reactions that cause the breakdown of organic matter. Breakdown of polymers such as carbohydrates, lipids, proteins occurs in four distinct stages (hydrolysis, acidogenesis, acetogenesis and methanogenesis) under the concerted activities of four different categories of microorganisms (hydrolytic bacteria, acidogens, acetogens and methanogens), resulting in the production of monomers (sugars, amino acids, fatty acids), then into other metabolites such as volatile fatty acids, aldehydes, alcohols and ultimately, methane, carbon dioxide and traces of other gases [45]. As the process goes through these different stages, the pH changes as the microorganisms perform particular functions, and the resulting end-products of each stage/phase serve as the substrates for the subsequent phase or stage. Existing as communities within the biodigester, the microbes can alter/modify the environmental pH as they consume resources and excrete metabolites. This environmental change has an impact on the growth and survival of the microbe and other microbial species co-habiting in the environment [51]. Compounds or substances present in a medium determine its pH, termed as its buffering capacity; therefore, the pH of the digesting substrate affects microbial dynamics and the stability of the anaerobic process. Considering that pH is vital to micro-organism’s survival, and can be easily measured, buffered and manipulated, pH can be considered a model environmental parameter, indicating acid accumulation that can lead to system failure [51].

In this study, the pH of the digesting medium was in the range 5.46 to 6.52, as shown in Figure 1. It was observed that *L*. *monocytogenes* had the highest viable counts in the slurry with pH 6.52 (that is prior to the commencement of the anaerobic digestion process). As the process progressed, pH declined due to degradation of the organic matter in the substrate, and there was a decrease in the viable counts. Rosso et al. [52] explained that environmental pH deviation from optimum pH levels causes a decrease in microbial growth rates because it modulates the thermodynamics and kinetics of redox reactions, thereby determining the structures of microbial communities. As the process continued, fluctuations occurred in the pH value and there was a further gradual decrease in the bacterial viable counts. A 1 log reduction in *Listeria* count occurred throughout the process until no growth was observed in *Listeria* selective Oxford agar environment inoculated with a dilution 10^−1^. Therefore, the viable counts registered were below the detection limit (i.e., <100 cfu/g) and the time taken for this was considered the survival period of the organism. Taking into consideration the findings of Manyi-Loh and Lues [31], who noted that *L*. *monocytogenes* had the longest survival time (175 days) compared to the other bacteria studies, this might suggest the bacterium had the potential to adapt to the varying pH occurring in the medium which decreased gradually over time. Adaptation of the bacterium to acid is critical to its survival, which explains its ability to persist in the food processing environment [53]. When *L*. *monocytogenes* grows in a mild acid environment, it can cause the organism to increase its resistance to pH that occurs at lethal levels in the stomach, i.e., an acid tolerance response. The acid tolerance of this pathogen can be associated with multiple acid resistance and the intracellular pH regulation systems, including the glutamate decarboxylase system, arginine deiminase system, and two regulatory and proton pumps (F_0_-F_1_-ATPase) [50], with these systems acting simultaneously to permit the survival and the adaptation of this bacterium to acid stress conditions. The regulation systems in cells permit the decarboxylation of amino acids (glutamate and arginine) via enzyme catalysed reactions, and regulate reactions that produce compounds with the potential to neutralise the low pH (ammonia production from urea or amine containing amino acids). In addition, proton pumps efflux protons out of the cell at the expense of ATP consumption, and the lipid composition of the cytoplasmic membrane is modified to lessen the permeability to protons [50]. Overall, microbes are exposed to different stresses relative to environmental stress, and they modify their optimum conditions for survival. In this light, Shamloo and colleagues [54] affirmed that the survival of *Listeria* at low pH is strongly dependent on low temperature.

Temperature functions as an essential factor in determining the efficiency of the anaerobic digestion process. Based on the temperature at which the process occurs, the anaerobic digestion of substrates can be grouped into thermophilic (50–70 °C), mesophilic (25–40 °C) and psychrophilic (below 25 °C) [45]. Accordingly, microorganisms are grouped into thermophiles, mesophiles and psychrophiles depending on the temperature at which the organism thrives and operates. In relation to biogas technology (anaerobic digestion), several findings have shown that the thermophilic and mesophilic temperature ranges are well suited for efficient biogas production and pathogen inactivation [55,56]. However, mesophilic anaerobic digestion is preferred to the thermophilic process because the latter is vulnerable to increase in odour production and process instability, and requires extra power [57]. Temperature is viewed as an essential factor, related to its effects on biological processes, including anaerobic digestion, since it affects microbial metabolic reactions carried out by enzymes; therefore, increasing temperature within a certain threshold (32–37 °C) leads to an increase in metabolic rate causing an increase in microbial growth and higher biogas yields. Temperature also affects other parameters that are relevant to an efficient anaerobic digestion process, including hydraulic retention time, ammonia formation, chemical oxygen demand, and the composition of the microbial communities [58,59]. In most cases, a small deviation from the optimum temperature range may result in a significant reduction in overall productivity and reproducibility of the process. In a biodigester, a bioengineered environment, the microorganisms are exposed to a changing environment of pH, nutrient availability, and increases or decreases in temperature [60]. 

Anaerobic digestion/co-digestion is considered as an important tool for the sanitisation of wastes via the inactivation of the zoonotic pathogens occurring in animal manure, and it is well documented that the rate of inactivation occurs faster with increasing temperature from the psychrophilic to the thermophilic temperature range [61]. In our study, we investigated the co-digestion of a blend of pig manure and pine wood sawdust over a period of seven months. As shown in Figure 2, the entire anaerobic digestion process operated in a temperature range of 18.4 to 29 °C over seven months. The first five months described the psychrophilic phase, and the latter two months constituted the mesophilic range. *Listeria monocytogenes* has the capacity to grow over a broad temperature range from −0.4 to 45 °C. Alteration in temperature can lead to several interconnected metabolic changes that are complex. These temperature changes can be indirectly detected by the organisms or by its specialised sensory systems, followed by adaptation of metabolic processes and responses, including adapted gene expression [60]. Akindolire and colleagues [57] critically evaluated the microorganisms and enzymes driving a psychrophilic anaerobic digestion process and noted a strong negative impact on microbial growth and enzyme activity. The authors further commented that psychrophiles have evolved with an array of genotypic and phenotypic adaptive features, enabling them to overcome barriers associated with cold or low temperature environments. The performance of an anaerobic digestion process depends on the concerted activities of all the groups involved in the process, although they have optimum growth rates at varying temperatures. Discrepancies in the sensitivity and resistance of the microbes to temperature stress conditions govern their thermal adaptation, and is the key factor influencing community structure and response [62]. The findings of Akindolire et al. [57] showed that the psychrophilic anaerobic digestion is conducted by cold-loving psychrophiles and cold adaptable psychrotrophs. Cold adaptable psychrotrophs are microbes exhibiting the ability to grow at temperatures below 15 °C but demonstrate maximum growth rate at temperatures above 20 °C. *Listeria monocytogenes* is a psychrotroph with the ability to live at refrigeration temperatures. For five months (the psychrophilic phase), the organism responded to cold temperature stress conditions in different ways, including stimulation of sigma factor protein, heightened accumulation of glycine betaine and carnitine from the surrounding via the chill activated transport system, and cells adapting by using two histidine kinases (*yycGF* and *lisRK* genes were identified) [63].

Throughout, the process was subjected to slight changes in temperature even within the two distinct phases. At the onset of the anaerobic digestion process, the temperature of the slurry was 18.4, the pH 6.52, and the *Listeria* counts was 1 × 10^4^ cfu/mL (log bacterial counts was 4.0). As the temperature dropped to 16.81, there was a simultaneous decline in bacterial counts to 9 × 10^2^ cfu/mL (log bacterial counts was 2.95). The mesophilic regimen occurred in the temperature range from 25.4 to 29 °C. With a sudden rise to 25 °C, a spike was observed in *Listeria* viable counts, which then decreased gradually until the end of the process, owing to the slight steady rise in temperature. After 175 days of anaerobic digestion, there was no growth in the 10^−1^ dilution sample as reported by Manyi-Loh and Lues [31]. So far, inactivation of bacterial pathogens via psychrophilic anaerobic co-digestion cannot be clearly attributed to a particular factor, but might be due to a combination of factors [31]. 

Osek et al. [63] showed that the presence of genetic factors permits the organism to adapt to physical and chemical factors by producing biofilms, colonizing, and persisting in the environment for a long time. A 100% recovery of the organism resulted in the identification of 43 *Listeria monocytogenes* strains from a pool of 74 presumed isolates. These findings corroborate those of Shourav et al. [64], who recovered *Listeria monocytogenes* in feed and dung samples procured from several cattle farms in Dhaka city, Bangladesh. According to the authors, the organism’s occurrence is critical because even at relatively low prevalence it has the potential to cause great mortality rates in both humans and animals should it navigate the food chain, especially through the consumption of contaminated dairy or meat products. In addition, the data should not be overlooked because their antibiotics characterisation was not known. The ubiquitous nature of this bacterium, added to its ability to form biofilms structures on various surfaces (which serves as a reservoir of contamination), creates difficulty in controlling and managing the organism, allowing it to persist for a long time, thereby allowing it to be repeatedly introduced into the food-producing environment with the potential to spread to other environments, including food and ultimately, humans [65].

Morbidity and mortality in low-income countries, most of which are found in Africa, are defined as Group I conditions, which include infections, maternal, perinatal and nutritional conditions. Govender and colleagues [66] stated that South Africans are living with the coexistence of undernutrition and overnutrition as a double burden of malnutrition expressed in terms of food and nutrition insecurity, poverty and unhealthy lifestyles, especially in pregnant women and children under 5 years, causing public health concerns. Regardless of food insecurity, South Africa contributes to the total increase in global meat consumption, indicating that it has a very high rate of meat consumption because of rising income, urbanisation and rapid population growth [67]. High meat consumption results in intensification of farming processes that use antibiotics. Accordingly, the South African Veterinary Association has provided guidelines endorsing the use of critically and highly important antibiotics, including streptomycin, gentamicin, erythromycin, ampicillin, ciprofloxacin and tetracycline in pig farming. These antibiotics are employed for the growth and welfare of the animals to augment productivity, but can lead to antibiotic resistance and zoonotic diseases. South Africa has a high disease burden and has experienced substantial effects on health and wellbeing due to HIV/tuberculosis, chronic illness and mental health, maternal and neonatal difficulties, child mortality, injury and violence [68]. Overall, the leading cause of death is HIV/AIDs, and the population living with HIV is prevalent and relies solely on the consumption of antimicrobials to treat immunocompromised systems [69]. Therefore, knowing the current trends in antimicrobial resistance is crucial. 

The threat of antimicrobial resistance is of great concern in developing countries, including South Africa, because it is associated with a high burden of infectious disease. The two major triggers of resistance seem to be unsuitable antibiotic therapy and extended use of antibiotics [70]. In many regions, antibiotics are overused and misused in people and animals, since they are purchased over the counter and consumed without a prescription from a professional. They are often used in infections not caused by bacterial organisms and are added to animal feed as growth promoters [71]. The association between antibiotic use in the livestock industry and meat production varies widely across the world. The frequencies and the quantities in which antimicrobials are employed in food production, are directly related to the possibility of the emergence of resistant foodborne pathogens [72]. 

Table 1 shows the profiles of sensitivity of each *Listeria* isolate to different antibiotics. The isolates responded differently to antibiotics belonging to different classes based on their chemical structure, i.e., aminoglycosides, amphenicols, penicillins, cephalosporins, macrolides, polypeptides, quinolones, sulphonamides, tetracyclines, nitrofurans and combined antibiotics that deliver their potency via different modes of actions, such as targeting the cell wall, inhibiting protein synthesis and DNA replication, or inhibiting folic acid metabolism [73]. Although serotyping of *L*. *monocytogenes* was not conducted in this study, our findings are supported by those of Acciari and colleagues [74], who studied the genetic diversity of *L*. *monocytogenes* strains from a food or food processing environment, and noted the occurrence of more than one serotype and pulsotype, yielding 2 to 23 *L*. *monocytogenes* isolates from a positive sample.

In our study, the *Listeria* species demonstrated remarkable (100%) susceptibility to gentamicin, sulfamethoxazole and ampicillin, which conforms to the 100% susceptibility to gentamicin noted by Shourav et al. [64] in their study. Troxler et al. [75] reported natural sensitivity or intermediate sensitivity to beta lactams (penicillins) and aminoglycosides. As a result, conventional therapy for the treatment of listeriosis in humans has been through the use of either penicillin or ampicillin as a single therapy or as a combined therapy in conjunction with an aminoglycoside (gentamicin) [76]. Of great significance is the marked sensitivity displayed to the antibiotics ampicillin and gentamicin recommended by SAVA for pig farming. This somewhat contradicts the pre-existing idea that exposure to antibiotics over a prolonged period (as in pig farming) can provoke or exacerbate antibiotic resistance. This is because Manyi-Loh et al. [22] published reported resistance to ampicillin (12.5–71.88%) and gentamicin (4.44–57.44%) by Gram-negative bacteria, including *E*. *coli*, *Salmonella* sp., *Yersinia* spp., and *Campylobacter* recovered from a co-digesting mixture of pinewood saw dust and pig manure. The discrepancies between these findings can be attributed to the differences in bacterial cell wall structures that confer different responses to external stresses such as antibiotics, heat, and UV radiation [77]. Clearly, the occurrence of a distinctive and protective structure, the outer membrane layer in the Gram-negative bacteria, distinguishes this group of bacteria from Gram-positive bacteria (e.g., *L*. *monocytogenes*). Breijeh et al. [78] explained that the outer membrane is responsible for the resistance of these bacteria to a wide range of antibiotics because the majority of the antibiotics, including Beta Lactams, quinolones, and colistins, amongst others, must travel through the outer membrane to reach their target sites. On the other hand, the *Listeria* isolates belonging to the Gram-positive category possess a thick peptidoglycan layer without a lipopolysaccharide outer membrane, facilitating the movement of cell wall active antibiotics to their respective sites of action, thus inhibiting, or killing, the bacterial cell [77].

The high susceptibility to both drugs (gentamicin and ampicillin) means that these antibacterial agents are not being used for treatment, or included as components of growth-promotion during fattening of animals. As a result, no selective pressure is exerted on the bacteria. High sensitivity was shown to cotrimoxazole (97.67%), augmentin (93.02%) and streptomycin (90.70%), suggesting these drugs, alongside sulfamethoxazole, gentamicin and ampicillin, are to be highly recommended in combating *Listeria* species. We tested the effect of antibiotics on *L*. *monocytogenes* with combined antibiotic formulations, including trimethoprim/sulphamethoxazole (also known as bactrim or co-trimoxazole in the concentration 23.75:1.25 µg per disc) and amoxicillin/clavulanic acid (also known as augmentin or amoxiclav in the concentration 20:10 µg per disc). These antibiotics are usually combined to produce a synergistic effect against the tested bacteria, thereby improving on the efficacy of the treatment. Ahmed et al. [79] defines synergy as the combined effects of two drugs being greater than the sum of their individual activities. This also reduces the likelihood of the development of antibiotic resistance, as it seems the chances of development of resistance against two antibiotic agents are lower compared to a single antibiotic [80]. In addition, Ahmed et al. [79] purported that antibiotic combination therapy increases or broadens the antibacterial spectrum. Similarly, Wang et al. [81] reported that a combination of antibiotics helped to reduce the dosage of one of the antibiotics that usually presented with serious side effects, thus lessening the chance and the occurrence of side effects. Combination therapy is vital for critically ill patients. *L. monocytogenes* is an intracellular pathogen, causing listeriosis as a serious health problem, and antibiotics must be transported into the host cells during treatment. Our study showed the potent activity of two combined antibiotics, co-trimoxazole (97.76%) and augmentin (93.02%) against *L. monocytogenes* isolates in an in vitro study. Cotrimoxazole is considered the drug of choice in patients who are allergic to penicillin. In comparing the activity of the combined antibiotic, amoxiclav, to that of the single antibiotic, amoxicillin, approximately 93% of the *Listeria* isolates demonstrated susceptibility to amoxiclav as opposed to sixty-seven (67%) of the isolates sensitive to amoxicillin as a monotherapy. Therefore, the *Listeria* isolates recovered in this study can be better treated with amoxiclav included in the empiric antibiotic treatment rather than amoxicillin, resulting in a 3% likelihood of antibiotic resistance compared to a 33% probability associated with amoxicillin. These findings contradict those of Sanlibaba et al. [38] who noted a 53% and 17.7% resistance to amoxiclav and cotrimoxazole, respectively, for their *L*. *monocytogenes* strains isolated from ready-to- eat food in Turkey; in addition, all the isolates were multidrug resistant.

Certain fractions (4.65–25.58%) of *L*. *monocytogenes* isolates demonstrated intermediate susceptibility to seven different antibiotics, including cefotaxime, ciprofloxacin, tetracycline, chloramphenicol, streptomycin, nalidixic acid and erythromycin. The categorisation of bacterial isolates into susceptible, intermediate and resistant is fundamental to antibiotic susceptibility testing [39]. The intermediate category is a grey zone and can be described as uncertain therapeutic success for the tested drug or drug combination, considering drugs whose dosing can be increased [82]. A striking implication of the intermediate sensitivity is that it helps to avoid critical errors in categorisation, owing to imprecise readings of zones of inhibition. According to Karlmeter [83], the intermediate category can be described as a buffer zone existing between the susceptible and the resistant zones, thus catering for little, uncontrolled and technical factors that might arise and cause discrepancies in the interpretation. However, it can also be termed as a therapeutic success in situations where the drug is able to accumulate at infection sites, or when a high dosage of drugs can be used, with a lower response rate compared to susceptible isolates [84]. 

Our results relating to intermediate sensitivity should not be discarded or considered as indicating resistance, since they may indicate possible, viable options for therapy. Owing to the development of antimicrobial resistance and the scarcity of new agents, the number of antimicrobial alternatives is declining. This necessitates the search for an agent considered sensitive, but this practice often targets the most potent drugs [83]. This situation must be avoided to prolong the life span and effectiveness of contemporary drugs. In this light, drugs with unequivocal intermediate sensitivity that can be concentrated at the site of the infection, or whose dosage can be increased, should be considered. 

Data on antibiotic resistance profiling of the isolated *Listeria* species presented in Figure 3 is a cause for concern from the environmental and public health perspective. Not only does surveillance of antimicrobial resistance provide an indication of the magnitude of current trends in the antibiotic resistance pattern, it can also help to evaluate the effectiveness of control measures that are being implemented to mitigate the crisis. The percentage resistance ranged from 0 to 51.16% of the different tested antibacterial agents that are commonly used in veterinary and human medicines. The observed resistance demonstrated by the *Listeria* species occurred in the order; nalidixic acid (51.16%), nitrofurantoin (48.83%), cefotaxime (44.19%) and amoxicillin (32.56%). This is in contrast with the finding of Sanlibaba et al. [38] who demonstrated total resistance (100%) to nalidixic acid of all the *L*. *monocytogenes* strains involved in their study. Interestingly, Troxler et al. [75] mentioned the natural resistance of *Listeria* species to older quinolones, especially nalidixic acid, demonstrating restricted activity against Gram-positive organisms, including *Listeria* species.

Although the resistance to amoxicillin (32.56%), erythromycin (25.58%), chloramphenicol (13.95%) and tetracycline (6.98%) recorded in this study was not very high, these are antibiotics of choice commonly employed for the treatment of human listeriosis. Therefore, the findings should not be overlooked as they indicate future resistance in *Listeria* species [19]. The bacterium acquires antibiotic resistance due to adaptive mechanisms that include efflux pumps, biofilm formation, and the exchange of antibiotic resistance traits with other species of bacteria via horizontal gene transfer, all resulting in the ineffectiveness of the antibiotics. In addition, the displayed resistance profile contradicts the patterns observed in other studies that investigated the antibiotic resistance profiles of the said organism, occurring in different environmental samples across different regions of South Africa [28,85,86,87]. Consequently, there seems to be a considerable variation in the occurrence of antibiotic resistance in *Listeria* species because of the continuous emergence of resistance patterns over the years and from different sources [15]. Klibi and colleagues [88] and Moreno et al. [13] remarked that the variation may be attributed to the use of numerous antimicrobials in several geographical territories in different periods.

According to Aarestrup [89] *Listeria* species are exposed to minute quantities of antibiotics, considering these substances are being employed in huge volumes in different activities related to human and animal life. Kimera and co-authors [90], as well as Selaledi and co-authors [91], described the frequent use of antimicrobials in food producing animals in Africa, for the enhancement of their growth and health. This practice has helped in the reduction of on-farm mortalities, reduction of the incidences of diseases and, above all, has resulted in improvement of productivity [91], thereby creating economic benefits to both the producers and consumers because the animals are reared for both food and as a source of income [92]. Nevertheless, the practice is viewed as the main contributor to the current crisis caused by antibiotic resistance [92]. This can be explained by the fact that the practice increases selective pressure, favouring the development of antibiotic resistance occurring via mutations or the acquisition of mobile genetic elements. Consequently, the findings of our current study are in line with several others [7,10] relating to antibiotic resistance in *Listeria* species found in environmental samples, and are not surprising. It is apparent that the use of antibiotics at sub-therapeutic levels in the mass production of poultry, eggs, and pork has promoted the development and maintenance of MAR organisms occurring in the faecal environment of these animals [41]. The appearance of antibiotic resistant bacteria in the food chain is amongst the underlying challenges encountered by the food industry. Bertsch et al. [93] purported that only the prevention and/or reduction of antibiotic treatment/prophylaxis in livestock will curtail the number of resistant microorganisms that are threatening human and animal health. 

The MAR index values ranged from 0 to 0.71, and eighteen isolates showed MAR values higher than 0.20, indicating these isolates were from a high-risk source where they had been exposed to antibiotics often. Quaik et al. [94] mentioned that veterinary antibiotics are excreted by animals in large amounts in their waste, including manure and urine. According to Agga and co-authors [95], the occurrence of antibiotic in animal manure can cause continuous selective pressure, resulting in the development of resistance to antibiotics. Our data further affirm the occurrence of multiresistant strains in nature, and the resulting serious threats to environmental and public health. Approximately, 41.86% of the *L*. *monocytogenes* isolates were multidrug resistant, demonstrating resistance to three or more antibiotics in the present study. Other authors have noted the varying prevalence of multidrug resistant *Listeria* species or *monocytogenes* across the globe, with 2.5% in Russia [10], 33.33% in Brazil [7], 54% in Spain [15], 73.91% in Turkey [14], and 100% in Bangladesh [64] and Malaysia [19]. These data indicate geographical differences, which could be associated with regional use of antimicrobials. 

### Antimicrobial Stewardship

Al-Omari et al. [96] defined antimicrobial stewardship as a collection of coherent actions either at the individual level, national level or global level from the human, the animal, and the environmental perspective, which will result in the promotion of prudent use of antimicrobials. Monitoring of antibiotic consumption, reassessment of prescribed antimicrobials after culture, conducting sensitivity reports, upholding surveillance of antimicrobial resistance, prescribing antimicrobials only when indicated, and using antimicrobials only for a brief period and on evidence, are amongst the core features of antimicrobial stewardship [96,97,98]. 

In addition, antimicrobial stewardship programmes emphasise antibiotic prescribing practices, strengthened by an understanding of the local antibiotic susceptibility trends, which in turn relies on the availability of a reliable medical microbiology laboratory resource [99]. Antimicrobial stewardship operates to optimise therapy, clinical outcome, reduces hospital costs and lengths of stay, as well as minimising the consequences of antibiotic resistance, since it takes into consideration the selection, dosage, and duration of the antimicrobial therapy throughout the course of use [96]. It is apparent that antibiotic resistant bacteria originating from environmental samples are not restricted only to the environment, but can eventually be channelled through the food chain into the clinical settings. Therefore, the findings of this study will go a long way to create an impact on antimicrobial stewardship and cannot be treated in isolation or ignored. This is because the One Health approach for the containment of antimicrobial resistance was postulated by the World Health Organisation to address antimicrobial resistance from clinical settings, the environment (animals, plants and soil) and food. 

Accordingly, antibiotic susceptibility testing performed by various techniques is the key procedure in determining the susceptibility of bacteria to antimicrobial agents, as well as to detect resistance in a clinical microbiology laboratory, thereby impacting on the choice of an appropriate antibiotic to be employed in the treatment of infections caused by a specific bacterium [39]. The intensity of the antibiotic action can be demonstrated via broth dilution tests, a disc diffusion test, or by using an automated instrument system [84]. In aggregate, findings based on characterisation into sensitive, intermediate and resistant categories can be assessed to provide the degree of resistance to each drug in a population. The degree of resistance reflects the quantity of antibiotic consumption. Results from sensitivity studies guide the choice of antibiotics to be used in the treatment of animals. Local surveillance data should guide the indication for the use, the choice, the dose and the duration of antibiotics in animal farming to improve on antimicrobial stewardship [98]. In this light, Huang et al. [97] and Al-Omari et al. [96] highlighted that the implementation of antimicrobial stewardship reduces antibiotic use and costs. 

Our study will serve as a baseline information for further studies into the organism serotypes and genes responsible for antibiotic resistance, as well as virulence traits.

## 5. Conclusions

This study indicates that swine manure is a potential carrier of pathogenic bacteria, capable of transmitting antibiotic resistance genes to humans. The prevalence (41.86%) of multidrug resistance in the isolates is perilous, especially for a pathogenic bacteria such as *L*. *monocytogenes* that has the capability to grow and thrive at refrigeration temperature and in other extreme environmental conditions occurring on an animal farm. The findings of this study emphasise the involvement of ampicillin and gentamicin drugs amongst first-line drugs for eradicating *Listeria* species or for empirical therapy, while advocating sulfamethoxazole to be a part of the empirical therapy necessary for the eradication of *Listeria* species. In addition, the findings from this study will help to update regional and national data on antibiotic sensitivity, resistance and multidrug resistance in *L*. *monocytogenes* isolates recovered from environment samples in South Africa, thereby influencing regional/nationwide policies guiding the prescription of antibiotics or the selection of appropriate therapy. The findings are of significance, as repeated antimicrobial susceptibility testing of an organism helps in monitoring clinical course, therapeutic success, and the emergence of resistance [83]. Furthermore, the data emphasise the need to seek for greener alternative approaches to maintain the health and the productivity of a herd, so that the effectiveness of antibiotics are sustained. Above all, our data are of relevance to public health leaders and specialists in infectious diseases, making it possible for them to detect emerging and novel resistance patterns. Similarly, this study emphasises the significance of continuous monitoring of the efficacy of the antibiotics recommended at this time, taking into consideration that a rapid change in resistance patterns might occur when the these antibiotics are overused. In addition, the findings emphasise the need to evaluate antibiotic resistance profiles on a local scale alongside global profiles, because of the inconsistency in antibiotic consumption in different geographical locations at different times, such that global patterns may not be a reflection of the local situation.

## Figures and Tables

**Table 1:** Frequency of varying susceptibility of *Listeria monocytogenes*.

Antibiotic Classes	Antibiotic Agents ^a^	Percentage Susceptibility of *L. monocytogenes* (%) ^b^		
		Susceptible (S)	Intermediate (I)	Resistance (R)
Aminoglycosides	Gentamicin (GEN)	100	0	0
	Streptomycin (S) ^c^	90.70	6.98	2.33
Amphenicols	Chloramphenicol (C)	74.42	11.63	13.95
Penicillins	Ampicillin (AMP)	100	- ^d^	0
	Amoxicillin (AML)	67.44	0	32.56
Cephalosporins	Cefotaxime (CTX)	30.23	25.58	44.19
Macrolides	Erythromycin (E)	67.44	4.65	25.58
Polypeptides Quinolones	Nalidixic acid (NA) ^c^	41.86	6.98	51.16
	Ciprofloxacin (CIP)	58.14	23.26	18.60
Sulfonamides	Sulfamethoxazole (SMX)	100	0	0
Tetracyclines	Tetracycline (TET)	79.07	13.95	6.98
Nitrofurans	Nitrofurantoin (NI)	51.16	0	48.84
Combinations	Augmentin (AUG) ^e^	93.02	0	6.98
	Co-trimoxazole (COT) ^e^	97.67	0	2.33

^a^ antibiotic tested agent; ^b^ number of isolated considered based on their measured diameter of zone of inhibition; ^c^ breakpoints for streptomycin and nalidixic acid were based on Enterobacteriaceae values; ^d^ no intermediate breakpoints, only sensitive or resistant (-); ^e^ combination antibiotics; COT, co-trimoxazole is composed of trimethoprim (1.25 µg) and Sulfamethoxazole (23.75 µg/disc), AUG is comprised of amoxicillin (20 µg) and clavulanic acid (10 µg/disc); AML, amoxicillin; CIP, ciprofloxacin; C, chloramphenicol; CTX, cefotaxime; E, erythromycin; NA, nalidixic acid; NI, nitrofurantoin; TET, tetracycline; GEN, gentamicin, SMX, sulfamethoxazole; AMP, ampicillin; S, streptomycin.

**Table 2:** Listeria isolates resistant to two or more antibiotics.

Number of Antibiotics	Number of Resistant Isolates	MAR Phenotypes
2	3	NA, NI
	1	E, C
	1	NA, E
3	2	NA, NI. AML
	1	NA, NI, CTX
4	1	NA, NI, AML, E
	1	NA, NI, E, CIP
	1	NA, NI, AML, CIP
	1	NA, NI, E, C
	1	NA, NI, AML, CTX
5	2	NA, NI, AML, CTX, TET
	2	NA, NI, AML, CTX, CIP
	1	NA, NI, CTX, CIP, E
	1	NA, NI AML, CTX, E
	1	NA, NI, CIP, E, C
6	1	NA, NI, CTX, E, C, AUG
9	1	NA, NI, AML, CTX, CIP, COT, E, C
10	1	NA, NI, AML, CTX, CIP, TET, AUG, E, C, S

AML, amoxicillin; AUG, augmentin; CIP, ciprofloxacin; C, chloramphenicol; COT, Co-trimoxazole; CTX, cefotaxime; E, erythromycin; NA, nalidixic acid; NI, nitrofurantoin; TET, tetracycline; GEN, gentamicin, SMX, sulfamethoxazole; AMP, ampicillin; S, streptomycin.

## Data Availability

Not applicable.
